# The role of surface chemistry-induced cell characteristics on nonviral gene delivery to mouse fibroblasts

**DOI:** 10.1186/1754-1611-6-17

**Published:** 2012-09-11

**Authors:** Tadas Kasputis, Angela K Pannier

**Affiliations:** 1 Biomedical Engineering Program, University of Nebraska-Lincoln, Lincoln, NE, USA; 2 Department of Biological Systems Engineering, University of Nebraska-Lincoln, 231 LW Chase Hall, Lincoln, NE, 68583-0726, USA; 3 Nebraska Center for Materials and Nanoscience, University of Nebraska-Lincoln, Lincoln, NE, USA; 4 Center for Nanohybrid Functional Materials, University of Nebraska-Lincoln, Lincoln, NE, USA

**Keywords:** Nonviral gene delivery, Transfection, Self assembled monolayers, Cell material interactions

## Abstract

**Background:**

Gene delivery approaches serve as a platform to modify gene expression of a cell population with applications including functional genomics, tissue engineering, and gene therapy. The delivery of exogenous genetic material via nonviral vectors has proven to be less toxic and to cause less of an immune response in comparison to viral vectors, but with decreased efficiency of gene transfer. Attempts have been made to improve nonviral gene transfer efficiency by modifying physicochemical properties of gene delivery vectors as well as developing new delivery techniques. In order to further improve and understand nonviral gene delivery, our approach focuses on the cell-material interface, since materials are known to modulate cell behavior, potentially rendering cells more responsive to nonviral gene transfer. In this study, self-assembled monolayers of alkanethiols on gold were employed as model biomaterial interfaces with varying surface chemistries. NIH/3T3 mouse fibroblasts were seeded on the modified surfaces and transfected using either lipid- or polymer- based complexing agents.

**Results:**

Transfection was increased in cells on charged hydrophilic surfaces presenting carboxylic acid terminal functional groups, while cells on uncharged hydrophobic surfaces presenting methyl terminations demonstrated reduced transfection for both complexing agents. Surface–induced cellular characteristics that were hypothesized to affect nonviral gene transfer were subsequently investigated. Cells on charged hydrophilic surfaces presented higher cell densities, more cell spreading, more cells with ellipsoid morphologies, and increased quantities of focal adhesions and cytoskeleton features within cells, in contrast to cell on uncharged hydrophobic surfaces, and these cell behaviors were subsequently correlated to transfection characteristics.

**Conclusions:**

Extracellular influences on nonviral gene delivery were investigated by evaluating the upregulation and downregulation of transgene expression as a function of the cell behaviors induced by changes in the cells’ microenvronments. This study demonstrates that simple surface modifications can lead to changes in the efficiency of nonviral gene delivery. In addition, statistically significant differences in various surface-induced cell characteristics were statistically correlated to transfection trends in fibroblasts using both lipid and polymer mediated DNA delivery approaches. The correlations between the evaluated complexing agents and cell behaviors (cell density, spreading, shape, cytoskeleton, focal adhesions, and viability) suggest that polymer-mediated transfection is correlated to cell morphological traits while lipid-mediated transfection correlates to proliferative characteristics.

## Background

Gene delivery approaches provide a mechanism to directly alter gene expression within a cell population. However, challenges with current delivery systems have limited the use of gene delivery for therapeutic, diagnostic, regenerative medicine, and tissue engineering applications. Nonviral vectors typically include cationic lipid or polymer-based systems capable of electrostatically complexing with negatively charged nucleic acids [1-3]. In contrast to viral-based gene delivery systems, nonviral vectors are more suitable for therapy given their lower toxicity and immune response, but are currently too inefficient to be considered relevant therapeutics [3-7]. This inefficiency is attributed to a number of extracellular and intracellular barriers that limit nonviral gene delivery. Extracellularly, mass transport limitations, vector cytotoxicity, complex degradation, and aggregation limit the ability of nonviral gene complexes to reach cells and be internalized [8]. Within the cell, intracellular processes such as endosomal escape, dissociation of the plasmid DNA from the chemical complexation vector, and nuclear entry of plasmid DNA limit transfection [8-10].

In order to overcome one or several of the barriers to nonviral transfection to improve gene transfer efficiency, most previous work has focused on physicochemical modifications of the complexing agent [11-14]. Other work has moved beyond the scope of physicochemical vector modifications and placed a focus on the extracellular microenvironment and its role in nonviral gene transfer [15-17], demonstrating that extracellular characteristics such as substrate stiffness, the presence of extracellular matrix (ECM) proteins, and RGD cell adhesion ligand density modulate nonviral gene transfer [18,19]. Furthermore, recent work has shown that ECM proteins immobilized to surfaces are capable of influencing the efficiency of nonviral gene delivery by modulating the cell cytoskeleton, endocytotic processes, and intracellular transport mechanisms [15,20].

However, most transfection studies have not addressed the role of cell-biomaterial interactions, in particular the effect of biomaterial surface properties that dictate cell behaviors that, in turn, could affect DNA transfer. Previous work has demonstrated that biomaterial surface characteristics such as surface chemistry (hydrophobicity, energy, end-functionalization), topography, and adsorbed protein density and conformation influence cell behaviors such as attachment, morphology, migration, proliferation, signaling, and differentiation [16,21-26]. Furthermore, while some previous studies have reported on the influence of cell-surface interactions on such cell behaviors, they have not determined statistical correlations between material properties and various cellular behaviors. Therefore, the objective of the present study was to examine the influence of cell-biomaterial interactions on nonviral transfection and, in particular, to link surface chemistry-induced cell morphological characteristics to gene transfer. In this study, statistical correlations were employed to determine significant correlations between cell-surface interactions and nonviral transfection characteristics. Self-assembled monolayers (SAMs) of alkanethiols on gold were used to provide surfaces with highly defined chemistries. SAMs provide smooth, well-defined surfaces with a high-degree of molecular order and packing [27,28]. SAMs have been shown to modulate cell-ECM interactions, which resulted in changes in cell density and spreading [29] and have been used to modulate cell-biomaterial interactions as well as to optimize the delivery of biomacromolecules [17,30-32].

Understanding the relationship between material surface properties and cellular response to gene delivery is essential in designing optimal material surfaces that can promote transfection through priming cells for transfection, without any alterations to delivery systems. The design of biomaterials with the ability to elicit specific cell transfection profiles is essential to improving both *in vitro* and *in vivo* applications, ranging from the development of substrates for *in vitro* culture studies, bioreactors, biotechnological assays, or functional genomics arrays, to the development of biomaterials for tissue engineering scaffolds that promote gene transfer.

## Results

### Surface modification

Substrate modification with SAMs was confirmed by measuring contact angles of water droplets in air on the SAM surfaces. The measured surface energy of -CH_3_ modified SAMs indicates the formation of hydrophobic surfaces (average contact angle of 95.3^o^ +/− 1.4), while -COO^-^ SAM surfaces were hydrophilic (average contact angle of 7.7^o^ +/− 1.0), as expected. Tissue culture polystyrene (PS), which contains high densities of carboxyl and hydroxyl functional groups exhibits a contact angle of 49^o^ +/− 5.3 and served as the control for all studies.

### Transfection

**Figure 1:**
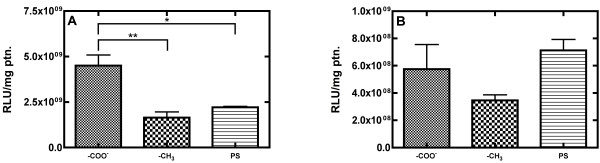
**Normalized transfection profile for cells plated on surfaces with defined surface chemistries transfected with (a) Lipofectamine 2000 and (b) PEI.** NIH/3T3 cells were seeded on SAM modified gold and tissue culture polystyrene control surfaces, nonviral DNA complexes with DNA encoding for EGFP and LUC were delivered 18 h later, and transfection profiles were acquired 48 h following complex delivery by quantifying the luciferase expression and normalizing these values per total protein amount present on the evaluated surfaces. Data is reported as mean +/− standard error of the mean of transfection profile values reported in relative light units (RLU) per mg protein. (*p < 0.05, **p < 0.01).

To examine the influence of surface chemistries described above on nonviral gene delivery, NIH/3T3 cells were seeded on SAM modified surfaces, nonviral DNA complexes were delivered 18 h later, and transfection profiles were acquired 48 h following complex delivery. Transfection was assayed through a combination of luciferase and Pierce bicinchoninic acid (BCA) assays, and transfection profiles were acquired by dividing the relative light units (RLU) of luciferase luminescence by the total mass of protein for each sample, which normalizes the degree of luciferase expression across the entire cell population for a sample. For transfection with Lipofectamine 2000, cells that adhered to charged, hydrophilic -COO^-^-terminated SAMs, were found to have a 2.7-fold increase of transgene expression in comparison to cells on uncharged, hydrophobic -CH_3_-terminated SAMs (Figure 1A, p < 0.01). Cells on -COO^-^- terminated surfaces also exhibited a 2.0-fold statistically significant (p < 0.05) enhancement of transgene expression relative to the PS control, whereas transgene expression of cells on -CH_3_-terminated SAMs was not significantly different from the PS control. Therefore, statistically significant increases in transfection efficiency were observed for cells seeded on –COO^-^ surfaces compared to polystyrene control surfaces. When PEI complexes were used to facilitate nonviral gene delivery, cells seeded on -COO^-^-terminated SAMs exhibited a 1.7-fold increase of transgene expression relative to -CH_3_-terminated SAMs (Figure 1B). Cells seeded on PS surfaces demonstrated the highest transfection levels in terms of the mean values of RLU/mg of protein for PEI transfection (Figure 1B). Although no statistically significant differences in transfection exist between the three surfaces evaluated using PEI, comparison of mean transfection profiles indicated differences in surface-induced transfection trends that were employed in subsequent statistical correlation analyses.

### Cell viability and proliferation

**Figure 2:**
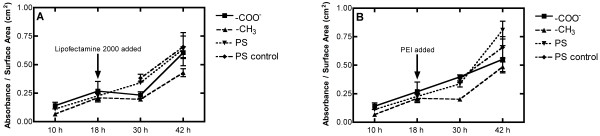
**Cell viability and proliferation measured with a MTT assay on surfaces with defined surface chemistries transfected with (a) Lipofectamine 2000 and (b) PEI.** The resulting absorbance was read at λ = 570 nm and results are reported as mean +/− standard error of the mean. The slope of the lines connecting each set of consecutive timepoints is indicative of the rates of cell proliferation as evidenced by the rate equation ((Δ absorbance/surface area)/Δ time).

An MTT assay was conducted to determine if the effect of surface chemistries on transfection could be attributed solely to cell viability and proliferation throughout the course of the transfection study. MTT production is considered to be a direct measure of a cell’s mitochondrial activity, although most researchers have attributed this measure of mitochondrial activity to an indirect measure of cell viability [33-36]. Cell viability was quantified for cells on the two different SAMs and the PS control at specific timepoints and proliferation rates were calculated between the evaluated timepoints similar to previous studies using MTT assay [37-39]. As seen in Figure 2, samples were collected at 10 h and 18 h following cell seeding. At the 18 h timepoint, nonviral complexes were introduced and these cells were analyzed 12 h and 24 h after nonviral complex delivery for viability and proliferation (30 h and 42 h after cell seeding). In addition, cells that were not treated with complexes on PS surfaces (PS control) served as the control for these studies. Prior to complex delivery, cell viabilities on the various surfaces were not statistically different (Figure 2 A and B) nor were the rates of cell proliferation ((Δ absorbance/surface area)/Δ time), depicted as the slope of a line connecting two consecutive timepoints.

Upon delivery of Lipofectamine 2000/DNA complexes, cells on SAM surfaces experienced slight decreases in cell proliferation rates, while cell proliferation rates on PS surfaces were unaffected, resulting in statistically significant increases in cell viability on PS surfaces relative to cells on SAM modified surfaces 12 h following the addition of Lipofectamine 2000 complexes (Figure 2A). At the final timepoint, 24 h following the introduction of Lipofectamine 2000 DNA complexes, there was an increase in the proliferation rate for cells on all surfaces when compared to their respective rates following the addition of complexes, with cells seeded on -COO^-^ SAMs demonstrating the highest proliferation rate, followed by cells on PS and -CH_3_ SAMs.

Cell viabilities in the presence of PEI complexes were statistically similar for all surfaces following complex delivery, with the exception of cells seeded on –CH_3_ surfaces immediately following complex delivery (Figure 2B). For studies conducted using PEI complexes, cells seeded on -CH_3_ surfaces experienced a decrease in cell proliferation rate upon being exposed to the PEI complexes while cells seeded on -COO^-^ and PS proliferated at similar rates as evidenced by MTT readouts 12 h following PEI-DNA complex delivery. Increased proliferation rates were demonstrated by cells seeded on all surfaces 24 h following PEI complex delivery, with the highest proliferation rate observed on PS surfaces.

### Cell morphology analysis

**Figure 3:**
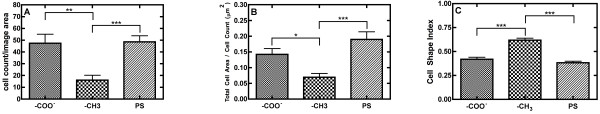
**(a) Cell density (b) cell spreading, and (c) cell shape index for cells plated on surfaces with defined surface chemistries.** Cell density was determined by counting the number of cells per image area, cell spreading was determined by measuring the total cell area per the amount of total cells per image area, and the cell shape index was determined by the equation S = 4πA/P^2^, where A is cell area, P is cell perimeter, and S is the cell shape factor. Data is reported as mean +/− standard error of the mean (*p < 0.05, **p < 0.01).

To further establish correlations between surface-induced cell behaviors and the extent of successful transgene expression, cell morphological characteristics – density, spreading, shape, cytoskeletal organization, and focal complex abundance – were analyzed on the three surfaces. Cell staining with Calcein AM and Hoechst dye was conducted 18 h following cell seeding on SAMs to determine the influence of different surface chemistries on cell density, spreading, and shape at the timepoint of transfection (data not shown). The number of adhered cells, referred to as cell density, was determined by counting the amount of nuclei of adhered cells per image using NIH ImageJ (Figure 3A). The average amount of adhered cells per image area was not statistically different between -COO^-^ SAMs and PS, whereas -COO^-^ SAMs and PS surfaces both exhibited 3.0-fold greater cell densities relative to -CH_3_ SAMs (Figure 3A, p < 0.01 and p < 0.001, respectively). Similarly, cell spreading was determined by measuring the total surface area of adhered cells normalized to the total cell count (Figure 3B). The degree of cell spreading was found to be greatest on polystyrene surfaces. In agreement with cell density trends, cells plated on -COO^-^ and PS surfaces had respective 2.1-fold (p < 0.05) and 2.7-fold (p < 0.001) increases in average cell surface area relative to cells plated on -CH_3_ SAMs. In order to further examine cell morphologies, individual cell shapes were analyzed on each SAM and PS (n = 150 for each surface). Cell shape indices (Figure 3C) were calculated using the cell shape factor, S, a dimensionless number ranging from zero to one, where a value of one indicates complete cell roundness and values closer to zero indicate less round cells [40]. Cell area and perimeter were measured for each cell using NIH Image J. Cells plated on -CH_3_ SAMs demonstrate significantly more rounded cell morphologies relative to cells on -COO^-^ SAMs and PS (p < 0.001), while no statistically significant differences in roundness were shown between cells on -COO^-^ SAMs and PS.

**Figure 4:**
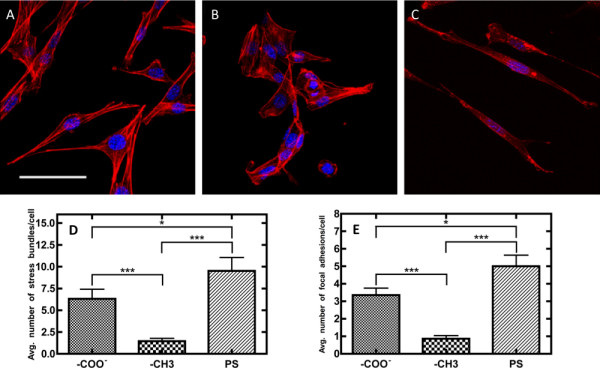
**Confocal microscopy images of NIH/3T3 fibroblasts stained with TRITC phalloidin for actin filaments (red) and nuclei counterstained with DAPI (blue) on (a) -COO**^**-**^**terminated, (b) -CH**_**3**_**terminated, and (c) PS surfaces.** Scale bar = 50 μm. Quantitative image analysis of (d) cytoskeletal stress bundles and (e) focal adhesions of NIH/3T3 fibroblasts on different biomaterial surfaces. Actin stress bundles were quantified by counting bundled actin fibers identified as bright regions of the TRITC stain, while focal adhesions were quantified by counting the amount of lamellipodial protrusions, which have previously been shown to directly correlate to focal adhesion abundance. Data is reported as mean +/− standard error of the mean (*p < 0.05, ***p < 0.001).

Qualitative and quantitative image analysis was performed on cells stained for f-actin filaments 18 h following cell seeding (Figure 4). Actin stress bundles were quantified by counting bundled actin fibers identified as bright regions of the TRITC stain [41], while focal adhesions were quantified by counting the amount of lamellipodial protrusions, which have previously been shown to directly correlate to focal adhesion abundance [42]. Quantitatively, the number of stress bundles (Figure 4D) and focal adhesion abundance (Figure 4E) were found to exhibit statistically significant differences for cells plated on all surfaces evaluated. Cells seeded on PS possessed the greatest amount of stress bundles and focal adhesions relative to cells on SAM surfaces, and cells on -CH_3_ SAMs demonstrated the lowest amount of these structures. Qualitatively, while cells seeded on -CH_3_ SAMs possessed the least amount of f-actin stress bundles, these cells still contained a considerable amount of individual f-actin fibers that were not as organized in contrast to the other two surfaces (Figure 4 A-C). Cells plated on both -COO^-^ SAMs and PS exhibited similar qualitative characteristics with a higher degree of f-actin filament organization (Figure 4 A and C) in contrast to cells on -CH_3_ surfaces (Figure 4B), as evidenced by the linear f-actin filaments traversing the entire lengths of these cells.

### Correlations of examined parameters

Correlations of cell parameters with surface functionalization procedures were determined by employing the Pearson’s product moment correlation equation [43]. A correlation matrix is presented in Table 1 containing the correlation coefficients for each comparison between cell behavior data sets; for example, a comparison between transfection trends among the surface variables and cell viability trends among the surface variables was investigated. Values approaching +1 signify a positive linear correlation, while values approaching −1 signify an inverse linear correlation, and where values approaching zero signify no correlation. As evidenced in Table 1, the correlations between transfection with Lipofectamine 2000 and cell morphological parameters (i.e. cell density, cell spreading, cell shape factor, cytoskeletal stress bundles, cellular focal adhesions) are not strong, while transfection with PEI is strongly correlated with the aforementioned cell morphological parameters since all correlation coefficients for these comparisons approach either −1 or +1, except for the correlation with cellular viability. Conversely, cellular viability is strongly correlated with transfection using Lipofectamine 2000 as evidenced by a correlation coefficient of 0.992 while the correlation between transfection with PEI and cellular viability is weak (0.443). As expected, all cell morphological parameters correlated strongly to each other, as evidenced by all correlation coefficients for these comparisons. The correlations between cell viability and the individual cell morphological characteristics varied, where both cell density and cell shape factor demonstrated modest correlations to cell viability and cell spreading, while cytoskeletal and focal adhesion characteristics correlate weakly to cell viability. The calculated correlation coefficients demonstrate that cell behaviors possess differing degrees of correlation with transfection characteristics based on the type of nonviral complexing agent.

**Table 1:** Pearson’s product moment correlation coefficient matrix calculated for comparisons between all measured parameters

	**Lipofectamine 2000**	**PEI**	**Cell Density**	**Spreading**	**Shape Factor**	**Cytoskeleton**	**Focal Adhesions**	**Viability**
**Lipofectamine 2000**	1.000							
**PEI**	0.325	1.000						
**Cell Density**	0.628	0.940	1.000					
**Spreading**	0.303	0.999	0.932	1.000				
**Shape Factor**	−0.522	−0.976	−0.992	−0.971	1.000			
**Cytoskeleton**	0.300	0.999	0.931	0.999	−0.970	1.000		
**Focal Adhesions**	0.302	0.999	0.932	0.999	−0.971	0.999	1.000	
**Viability**	0.992	0.443	0.722	0.422	−0.626	0.419	0.421	1.000

## Discussion

Nonviral gene delivery as a function of underlying surface chemistry was investigated to elucidate cell-surface interactions that could contribute to improving gene transfer, allowing for the design of cell microenvironments capable of enhancing nonviral gene delivery. Furthermore, understanding cell-surface interactions and their relation to nonviral gene delivery provides valuable information regarding the extracellular influences on nonviral transfection processes.

Nonviral gene delivery was evaluated in cells seeded on surfaces with defined surface chemistries. SAMs of alkanethiols on gold were used as model biomaterial interfaces since they provide smooth surfaces with defined surface chemistries that have been widely utilized to investigate cell-surface interactions [26,29,44-46]. Two different surface chemistries, negatively charged hydrophilic surfaces consisting of terminal carboxyl groups and uncharged hydrophobic surfaces consisting of methyl terminations, were investigated and transfection studies were conducted using two commercially available complexing agents. The reported transfection trends suggest that the two complexing agents interact differently with the cells attached to these surfaces, which could be influencing subse-quent complex internalization, intracellular trafficking mechanisms, and nuclear localization. Alternatively, the differences in transfection may also be explained by interactions with the complexes with the surfaces themselves, which was not investigated here, but previous work regarding nonviral complex interactions with surfaces and cells indicates that complexes interact electrostatically with charged surfaces [17] and non-specifically with adsorbed ECM and serum proteins, as well as binding to cell surface proteoglycans prior to endocytosis [47]. Therefore complex-surface interactions, in particular for DNA complex delivery to cells on negatively charged surfaces, could potentially be enhancing transfection. However, dramatic changes in cellular characteristics were observed on the different surfaces; therefore these characteristics were further investigated for their influence on nonviral gene transfer and statistically correlated to the transfection profiles obtained using polymer-based and lipid-based complexing agents.

Cell viability and the rate of cell proliferation were investigated because proliferation is commonly associated with successful DNA complex uptake and nuclear localization due to the compromised integrity of nuclear membranes of dividing cells [6,48]. During the course of transfection studies, cell viability was quantified for cells on the two different SAMs and the PS control at specific time points. Throughout the course of the viability studies, cell viabilities were not statistically different for the different surfaces for most of the assayed time points, which has been shown in certain cells seeded on SAMs [24,49]. Interestingly, cell viability trends demonstrated a modest correlation with cell density trends (Table 1), which suggests that surfaces not only induce differences in cell density, but that different surface chemistries induce differences in cell metabolisms, which in turn could be affecting transgene expression. Analysis of cell viability 12 h after delivery of Lipofectamine 2000 or PEI complexes did reveal a slight cytotoxic effect in the presence of these transfection reagents, in agreement with other studies that have demonstrated vector cytotoxicity [3,8,50]. Cells on SAM surfaces experienced a greater degree of cytotoxicity in the presence of nonviral complexes in contrast to the PS control, which may be due to a higher local concentration of complexes on SAM surfaces around cells. Evaluation of cell viability following the addition of nonviral complexes revealed a correlation between transfection levels and cell viability in the presence of Lipofectamine 2000 complexes (Table 1). However, for PEI complexes, transfection trends could not be attributed to cell viability (Table 1). Since distinct morphological differences were observed on the investigated surfaces, surface-induced cell morphologies and corresponding intracellular cytoskeletal and adhesive proteins, which are commonly linked to biomolecular import and subsequent intracellular trafficking, were investigated.

Cell density, spreading, and shape have been previously shown to be influenced by cell-surface interactions [21,29,44,51]. In the context of this study, we demonstrated a similar influence, but also demonstrated a statistical correlation between these characteristics and the success of gene delivery using PEI. First, the cell density was found to be most prominent on -COO^-^ SAMs and PS surfaces, while the cell density on -CH_3_ SAMs was significantly less, which strongly correlated with transfection trends (Table 1). Although a strong correlation was observed between cell density and transfection characteristics, the increase in transfection levels could not be simply correlated to cell number, as normalized transfection results were reported. Instead, this increase in cell density is indicative of surfaces that are conducive to cell adhesion, which is attributed to the subsequent morphological attributes that were evaluated. Increased cell adhesion and spreading is likely due to an increased abundance of cell adhesion domains of ECM proteins on the surface resulting in increased cell integrin binding to natively deposited ECM proteins as suggested by previous literature [25]. In addition to these phenotypic observations, our group has previously shown that transfected cells express upregulated levels of an endogenous gene, Rap1a, that has been implicated in integrin-mediated cell adhesion [52]. Second, for cell spreading, cells seeded on PS and -COO^-^ surfaces possessed an increased spreading area in comparison to cells seeded on -CH_3_ SAMs, which suggests increased exposure to nonviral complexes on PS and -COO^-^ surfaces. Third, congruent to cell spreading results, cell shape was found to be significantly different for cells seeded on -CH_3_ surfaces in comparison to cells on the other two surfaces. Cells on -CH_3_ surfaces were statistically more round, while cells on the other two surfaces demonstrated more spread and ellipsoid morphologies, analogous to cells that were adhered well to surfaces. Also, it is important to note that while cells on -CH_3_ surfaces were more round, they still remained viable as evidenced by the cell viability data (Figure 2) and live cell staining with Calcein AM (data not shown). Similar to correlations between transfection and cell adhesion, cell spreading demonstrated a strong correlation with PEI transfection and cell shape factor exhibited a strong inverse correlation with PEI transfection, which suggests that gross morphological characteristics significantly influence transfection with PEI. Since cell shape is manifested by various intracellular mechanisms such as cytoskeletal f-actin filament polymerization and organization, as well as the formation of focal adhesions, intracellular morphological characteristics were subsequently evaluated to examine possible influences of intracellular mechanisms on nonviral gene delivery.

F-actin stress bundles and focal adhesions were evaluated to examine intracellular phenomena that have been previously shown to be influenced by cell-surface interactions [53-55]. Focal adhesions and cytoskeletal characteristics are the result of cellular processes that are influenced by micro environmental stimuli and are involved with cellular interactions at surfaces [21,54-56]. Cytoskeletal and focal adhesion characteristics were evaluated in the context of nonviral gene delivery because these features have previously been shown to influence internalization mechanisms responsible for the internalization of nonviral complexes [10,20]. Cytoskeletal stress bundle (polymerized f-actin filaments) abundance and organization, as well as focal adhesion abundance, were found to be greatest in cells seeded on –COO^-^ and PS surfaces, and these results correlate strongly with PEI transfection results. The abundance of f-actin stress bundles has previously been shown to be directly correlated to the cytoskeletal tension in cells, which is important in the context of nonviral gene delivery because cytoskeletal tension influences cell shape and various endocytic processes [41], which have previously been shown to enhance biomolecule internalization [47,57]. In addition to stress bundle analysis, qualitative observations of individual f-actin cytoskeletal fiber quantity and organization reveal the greatest amount of polymerized f-actin cytoskeletal fibers and highest degree of fiber organization in cells seeded on PS and -COO^-^ SAMs. The abundance of focal adhesions also indicates that cells are more firmly anchored to surfaces, which has been shown to directly correlate to cell adhesive forces [21,56,58]. These observations and conclusions are supported by previous studies that have shown changes in cell cytoskeletal structure along with focal adhesions affecting macromolecule internalization pathways [47], therefore affecting the internalization of nonviral complexes [57].

The combination of the gross and fine cell morphological characteristics examined in this study directly correlate to transfection trends for cells seeded on surfaces with defined surface chemistries and transfected with PEI, and thus indicate that cell morphologies influence gene transfer. The correlations between cell characteristics and PEI transfection may be indicative of complex trafficking within vesicles that transport along cytoskeletal components that constitute these morphologies. However, the lack of correlation for Lipofectamine 2000 transfection with cell morphological factors suggests there may be other factors dictating its gene delivery success. Correlations between Lipofectamine 2000 transfection characteristics, proliferation rates, and certain cell morphologies to a minor degree, regulated through surface chemistries, suggest alternative trafficking mechanisms or enhanced nuclear transport in contrast to PEI-DNA delivery.

## Conclusions

Cell characteristics on model biomaterial surfaces were investigated in the context of nonviral gene delivery to elucidate the association between surface chemistry, cell morphological characteristics, and resultant transfection profiles. Cells adhered to negatively charged hydrophilic SAMs exhibited statistically significant enhanced transfection profiles for Lipofectamine 2000, while cells seeded on uncharged hydrophobic SAMs demonstrated statistically significant lower transfection with both polymer- and lipid-based DNA complexes. For PEI-mediated DNA delivery, transfection levels were high in cells on both negatively charged hydrophilic SAMs and PS, when compared to hydrophobic SAMs. In general, surfaces with high cell densities, more cell spreading, more cells with ellipsoid morphologies, and increased quantities of focal adhesions and cytoskeleton features within cells, statistically correlate with higher transfection profiles than surfaces that do not promote such cellular characteristics for polymer-mediated transfection. Conversely, cell viabilities at the time point of nonviral complex introduction correlate with the transfection profiles for lipoplexes. In future studies, additional surface characteristics including nanotopography, as well as the role of cell-surface interactions on complex internalization and endogenous gene expression profiles that may influence gene delivery will be investigated employing different cell types. Understanding the cell-biomaterial interface as it pertains to enhancing transfection efficiency heralds the development of precisely tunable substrates for biotechnological and tissue engineering applications.

## Materials and methods

### Surface preparation

Gold-coated slides were prepared by electron beam evaporation of gold (50 nm thickness) over a titanium adhesion layer (5 nm thickness) onto standard glass microscope slides (VWR International, Radnor, PA) and stored under argon gas. Prior to beginning experiments, the gold slides were cut into small square pieces using a diamond-tipped cutting instrument to allow the pieces to fit into wells of a 48-well cell culture plate. For SAM preparation the gold surfaces were cleaned with a copious rinse of acetone immediately followed by rinsing with an excess of degassed, sterile-filtered 200 proof ethanol and dried with nitrogen gas. The gold-coated surfaces were then immersed in 2.00 mM solutions of alkanethiol dissolved in degassed, sterile-filtered 200-proof ethanol. Carboxyl-terminated surfaces (−COO^-^) were formed by immersing gold surfaces in 11-mercapto-undecanoic acid (Sigma-Aldrich, St. Louis, MO, USA) for 45 min and methyl-terminated surfaces (−CH_3_) were formed by immersing gold surfaces in 1-decanethiol (Sigma-Aldrich) overnight (18–24 h). The samples were removed from their respective alkanethiol solutions and rinsed with copious amounts of ethanol to remove excess alkanethiol solution and dried with nitrogen gas. Unmodified tissue culture polystyrene (PS) well plate surfaces served as the control for all studies.

### Contact angle

Successful SAM surface modification was confirmed by contact angle measurements. Immediately following SAM formation, SAM surfaces along with unmodified PS controls were allowed to equilibrate in phosphate buffered saline (PBS) for 15 min and then angles of nanopure water (18.3 MΩ) were measured by the sessile drop technique using a goniometer (Ramé-Hart Instrument Co., Netcong, NJ).

### Cell culture

NIH/3T3 mouse fibroblasts (ATCC, Manassas, VA, USA) were used for all studies. Cells were cultured in Dulbecco’s Modified Eagle’s Media (DMEM, ATCC) containing 10% calf serum (Colorado Serum Co., Denver, CO, USA) and 1% penicillin/streptomycin (Invitrogen, Carlsbad, CA) and incubated at 37°C, 5% CO_2_. Prior to seeding cells, 200 μL of sterile-filtered 1X PBS (pH 7.4) was added to each well to equilibrate the SAMs at physiological pH. After 15 min, PBS was aspirated and 300 μL of NIH/3T3 cell suspension was added to each well at a seeding density of 50,000 cells/mL (15,000 cells/well) for all surfaces under investigation.

### Transfection

Plasmid *pEGFP-LUC*, which encodes for both the enhanced green fluorescent protein (EGFP) and firefly luciferase protein (LUC) under the direction of a cytomegalovirus (CMV) promoter (Clontech, Mountain View, CA), was used for all transfection experiments. Plasmids were purified from bacterial culture using Qiagen reagents (Valencia, CA) and stored in Tris–EDTA buffer solution (10 mM Tris, 1 mM EDTA, pH 7.4) at −20°C. After allowing seeded cells to adhere for 18 h, either polymer or lipid complexes containing a concentration of 0.3 μg of plasmid DNA per cell culture well were added to the well plates. Lipoplexes were prepared with Lipofectamine 2000 (Invitrogen) using a Lipofectamine 2000/plasmid DNA ratio of 1.75:1 in serum-free OptiMEM media (Invitrogen) according to manufacturer’s protocols. Complexes were allowed to form for 20 min at room temperature and then 75 μL of lipoplex solution was added drop-wise to each well undergoing lipofection. Polyplexes were prepared with 25 kDa branched polyethylenimine (PEI, Sigma-Aldrich) at a nitrogen/phosphorus (N/P) ratio of 15 in Tris-buffered saline (TBS) [59]. Upon combining the constituent materials, the polyplex solution was vortexed for 10 sec and polyplexes were allowed to self assemble for 15 min at room temperature. Then, 50 μL of polyplex solution was added drop-wise to each well undergoing PEI transfection. Following the addition of complexes, cell culture plates were placed in an incubator at 37°C, 5% CO_2_ for 48 h. Complexation conditions were optimized for both vectors on tissue culture PS prior to beginning transfection studies.

### Assessment of transfection

Fluorescence microscopy was conducted at 48 h following delivery of complexes to confirm the successful expression of the EGFP protein using a Leica DMI 3000B fluorescence microscope (Leica Microsystems GmbH, Wetzlar, Germany).

Following microscopic confirmation of transfection, gold chips were transferred into new wells and cells were lysed with 200 μL 1X reporter lysis buffer (Promega, Madison, WI). Transfection levels were quantified by measuring the luciferase activity using the Luciferase Assay System (Promega) and a luminometer (Turner Designs, Sunnyvale, CA). Luciferase activity (measured as relative light units, or RLUs) was normalized to the total protein amount determined with a Pierce BCA protein assay (Pierce, Rockford, IL). Each transfection experiment was performed in triplicate wells on duplicate days.

### Cell viability and proliferation

The influence of SAM surface chemistry on cell viability and proliferation was examined using a Vybrant® MTT Cell Proliferation Assay Kit (Invitrogen) according to the manufacturer’s protocols, modified by adjusting reagent volumes to accommodate assaying in 48-well plates. Cell viability was evaluated both prior to the addition of DNA complexes (10 h and 18 h following cell seeding) as well as 12 h and 24 h following the addition of DNA complexes (30 h and 42 h after cell seeding, respectively). The assay was quantified using a DU730 Life Science UV/VIS Spectrophotometer (Beckman Coulter, Brea, CA, USA) at λ = 570 nm and all readings were normalized against the surface area of each SAM or PS surface. Sample surface areas were obtained by taking photographs of the gold chips prior to SAM surface modification and subsequently measuring the surface areas with ImageJ (NIH).

### Cell morphology analysis

In order to analyze the influence of SAM surface chemistry on cell density, spreading, and shape, the cytoplasm of cells seeded on these surfaces were stained at the time point immediately prior to the introduction of DNA complexes. In accordance with manufacturer’s protocols, 2.00 μM Calcein AM (Invitrogen) diluted in serum-free cell culture media was added to each well and allowed to incubate at 37°C, 5% CO_2_ for 10 min. Then, the cell lawn was rinsed with 1X PBS followed by fixation in 300 μL of 10% neutral buffered formalin (Thermo Fisher Scientific, Waltham, MA) for 20 min at room temperature. Formalin was removed and cell nuclei were counterstained with Hoechst stain (1.0 μg/mL, Invitrogen) diluted in serum-free cell culture media and incubated at 37°C, 5 CO_2_ for 8 min. Following counterstaining, cells were rinsed with 1X PBS and imaged with a Leica DMI 3000B fluorescence microscope. For cell density and spreading image analysis, three images per well were acquired using a fluorescence microscope with a 10x objective and subsequently analyzed using NIH Image J. Cell density data were obtained by counting the amount of nuclei of adhered cells per image. Cell spreading quantification was accomplished by measuring the total surface area of adhered cells normalized to total cell count, thereby obtaining an average cell surface area. Images for cell shape index analysis were acquired at a 40x magnification and five images per well were analyzed to obtain statistically relevant n-values of cells (n > 150 cells). To obtain a quantification of cell shape, perimeters and areas of individual cells were measured using NIH Image J and the cell shape index was calculated using the formula S = 4πA/P^2^, where A is cell area, P is cell perimeter, and S is the cell shape factor [40].

To examine the effect of SAM surface chemistry on intracellular filamentous actin and focal adhesion complexes, cell staining and image analysis were conducted 18 h following cell seeding, the time point immediately prior to introduction of DNA complexes. Upon preparing SAMs, samples were seeded with cells as described above and allowed to adhere for 18 h. Then, cell culture media was removed and the samples were fixed with 4% paraformaldehyde (Electron Microscopy Sciences, Hatfield, PA, USA) in 1X PBS for 20 min. An Actin Cytoskeleton/Focal Adhesion Staining Kit (Millipore, Billerica, MA, USA) was used according to manufacturer’s protocol. Briefly, cytoskeletal filamentous actin (f-actin) fibers were stained with TRITC-conjugated phalloidin, and DAPI was used to counterstain cell nuclei. Following the actin staining procedure, the surfaces were mounted facedown onto glass cover slips using antifade mounting solution (Millipore). Samples were imaged using an Olympus FV500 Laser Scanning Confocal Microscope (Olympus, Shinjuku, Tokyo, Japan). F-actin fiber length and abundance of f-actin stress fibers and focal adhesions were quantified using ImageJ (NIH). Fifteen images per condition were evaluated (Five images per triplicate well).

### Statistics

All experiments were performed in triplicate wells on duplicate days. Statistical analysis was performed using Prism software (GraphPad Prism 5, LaJolla, CA). Mean values with standard error of the mean are reported for all numerical analysis. Comparative analyses were completed using one-way ANOVA with Tukey post-tests at a 95% confidence level (α = 0.05). Pearson’s product moment correlation coefficients were calculated [43] for comparisons between all measured parameters: transfection with Lipofectamine 2000, transfection with PEI, cell density, cell spreading, cell shape factor, cytoskeletal stress bundles, focal adhesion abundance, and cell viability at 18 h – the timepoint of nonviral complex introduction to cells.

## Competing interests

Both authors declare that they have no competing interests.

## Authors’ contributions

Tadas Kasputis, graduate assistant, formulated ideas surrounding the project, designed and conducted all experiments described in this manuscript, and is the primary author of the submitted manuscript. Dr. Angela K. Pannier is the principal investigator of this study and formulated the main ideas, hypotheses, and experimental design. Dr. Pannier was also involved in the writing and proofreading of the manuscript. Both authors read and approved the final manuscript.
